# Psychosocial impact of the summer 2007 floods in England

**DOI:** 10.1186/1471-2458-11-145

**Published:** 2011-03-03

**Authors:** Shantini Paranjothy, John Gallacher, Richard Amlôt, G James Rubin, Lisa Page, Tony Baxter, Jeremy Wight, David Kirrage, Rosemary McNaught, Palmer SR

**Affiliations:** 1 Department of Primary Care and Public Health, Clinical Epidemiology Interdisciplinary Research Group, School of Medicine, Cardiff University, (5th Floor Neuadd Meirionnydd, Heath Park) Cardiff (CF14 4YS) UK; 2 Emergency Response Department, Health Protection Agency, (Building H11, HPA, Porton Down), Salisbury (SP4 0JG) UK; 3 King's College London, Institute of Psychiatry, (3rd Floor, Weston Education Centre, 10 Cutcombe Road), London (SE5 9RJ) UK; 4 NHS Doncaster and Doncaster Metropolitan Borough Council, (White Rose House, Ten Pound Walk), Doncaster (DN4 5DJ) UK; 5 Sheffield Primary Care Trust and City Council, NHS Sheffield, (722 Prince Of Wales Road), Sheffield (S9 4EU) UK; 6 Health Protection Agency, (Elgar House, Green Street, Kidderminster), Worcestershire, (DY10 1JF) UK; 7 South Yorkshire Health Protection Unit, Health Protection Agency, (Meadowcourt, Hayland Street), Sheffield (S9 1BY) UK

## Abstract

**Background:**

The summer of 2007 was the wettest in the UK since records began in 1914 and resulted in severe flooding in several regions. We carried out a health impact assessment using population-based surveys to assess the prevalence of and risk factors for the psychosocial consequences of this flooding in the United Kingdom.

**Methods:**

Surveys were conducted in two regions using postal, online, telephone questionnaires and face-to-face interviews. Exposure variables included the presence of flood water in the home, evacuation and disruption to essential services (incident management variables), perceived impact of the floods on finances, house values and perceived health concerns. Validated tools were used to assess psychosocial outcome (mental health symptoms): psychological distress (GHQ-12), anxiety (GAD-7), depression (PHQ-9) and probable post-traumatic stress disorder (PTSD checklist-shortform). Multivariable logistic regression was used to describe the association between water level in the home, psychological exposure variables and incident management variables, and each mental health symptom, adjusted for age, sex, presence of an existing medical condition, employment status, area and data collection method.

**Results:**

The prevalence of all mental health symptoms was two to five-fold higher among individuals affected by flood water in the home. People who perceived negative impact on finances were more likely to report psychological distress (OR 2.5, 1.8-3.4), probable anxiety (OR 1.8, 1.3-2.7) probable depression (OR 2.0, 1.3-2.9) and probable PTSD (OR 3.2, 2.0-5.2). Disruption to essential services increased adverse psychological outcomes by two to three-fold. Evacuation was associated with some increase in psychological distress but not significantly for the other three measures.

**Conclusion:**

The psychosocial and mental health impact of flooding is a growing public health concern and improved strategies for minimising disruption to essential services and financial worries need to be built in to emergency preparedness and response systems. Public Health Agencies should address the underlying predictors of adverse psychosocial and mental health when providing information and advice to people who are or are likely to be affected by flooding.

## Background

Natural disasters, including flooding have been reported to have a wide range of psychosocial and mental health impacts, including psychological distress, anxiety, depression, somatisation and post traumatic stress disorder (PTSD) [1-5]. Following the tsunami in Southern Thailand and Hurricane Katrina in the USA, elevated levels of PTSD were reported in affected populations [6,7].

Known risk factors for poorer mental health following natural disasters include female gender, older age, lower educational achievement, lower household income, long term health problems and lower social support in the form of networks of family and friends [7-11]. Disaster specific variables such as evacuation and damage to property have also been identified as risk factors for mental ill health in some affected populations [9,12].

The summer of 2007 was the wettest in the UK since records began in 1914, resulting in severe floods and "the biggest civil emergency in British history" [13]. A Government Review commissioned following the floods (the Pitt review) reported that over 55,000 properties were flooded, around 7,000 people were evacuated and 13 people died [13]. The country also experienced the largest loss of essential services since World War II with almost half a million people without mains water or electricity. The estimated monetary cost of the floods to the insurance industry was in excess of £3 billion, not including other substantial costs that were met by central government, local private bodies, businesses and individuals [13]. Although worldwide there were over 200 floods affecting 180 million people, 8,000 deaths and over £40 billion in damages in 2007, the floods in England were the most expensive in the world in that year [13]. The Pitt review commissioned a survey of 647 households; 39% of respondents said the flooding had affected their physical health and 67% their emotional health [13]. A better assessment and understanding of psychosocial and mental health risk factors associated with emergencies is important for improved preparedness and mitigation [14,15]. Therefore, as part of the public health response to the floods in England in 2007 the Health Protection Agency and local public health departments carried out a health impact assessment.

In this paper we compare the prevalence of four mental health symptoms (psychological distress, generalised anxiety disorder, depression and post-traumatic stress disorder) between flooded and non-flooded populations, and examine the role of risk factors, particularly incident management variables (evacuation and disruption to essential services), as these can be modified or addressed in the future to minimise the psychosocial impacts associated with flooding.

## Methods

### Survey population

In June 2007, two areas were particularly badly affected, South Yorkshire and Hull, followed by Worcestershire, Gloucestershire and Thames Valley, in July 2007. The HPA health impact assessments were conducted in South Yorkshire (September - October 2007) and Worcestershire (January - February 2008). These areas were chosen because of the feasibility of carrying out the surveys. The timing of the surveys after the floods varied (3 months after the event in South Yorkshire and 6 months after the event in Worcestershire) due to the logistics of obtaining sampling frames and getting the surveys rolled out.

Local public health staff identified areas that had homes affected in varying degrees by the floods and homes that were not affected. The non-affected households were included as controls. In South Yorkshire, the sampling frame was all addresses in one housing estate (n = 347, population 1,500), all addresses in one village (n = 436, population 1,013), all addresses on the Local Authority flooded properties register in one town (n = 626), and a random selection of 1,252 addresses that were not on the flooded properties register. The town had about 5,000 households, population 12,000.

In Worcestershire, we surveyed all addresses in 2 villages (n = 460), all addresses on the Local Authority flooded properties register in two towns (n = 533) and a random selection of 7,995 addresses (of a total of 12,500) that were not on these registers. As the towns were much larger than the villages in terms of population size (29,000 in the towns compared with 950 in the villages), we sampled 15 properties not on the flooded register for every property that was on the flooded register.

### Data collection

A letter of invitation to participate in the survey was sent out to 'the occupier' of households by the Directors of Public Health for the area, outlining a choice of three methods to complete the survey, (i) by telephoning a free phone number and completing the questionnaire on the telephone, or (ii) by completing the questionnaire online or (iii) by return of a paper questionnaire using a freepost envelope that was provided. There was no restriction on who should complete the questionnaire. The questionnaire explained the reason for collecting the data and confirmed that any information provided would be treated with the strictest confidence and provided assurance that participation in the survey was voluntary and would not affect the healthcare received in any way. Due to the low response rate in South Yorkshire to the above three methods of data collection the decision was taken to use face-to-face interviews in the two villages in Worcestershire as these areas were small enough for this method of data collection to be feasible. The invitation letter sent to the two Worcestershire villages explained that data collection would be carried out in face-to-face interviews by interviewers who would visit the houses in the area the following week. Reminder postcards were sent out to non-responders after three weeks.

### Exposure variables

We recorded flooding (the presence of water in (i) street or garden outside the house; (ii) basement or cellar of the property or below floor level in the ground floor rooms; (iii) above floor level in ground floor rooms), and damage to property (possessions, furniture, kitchen units, documents or decoration or structures).

### Psychological exposure

We measured perceived impact of the floods using a five point likert scale of "much better, better, the same, worse, much worse" in response to the following questions: (i) As a result of the flooding how do you expect your financial circumstances to be? (ii) As a result of the flooding, how do you expect house values will be? We also asked about health concerns relating to the flood: 'Do you have any health worries relating to the flood for yourself or your family?'

### Incident management variables

The questionnaire included questions about disruption to essential services (such as gas, electricity and water supply) and evacuation (request to evacuate, a request made and refused, a request made and complied with).

### Outcome measures

Psychosocial impact was assessed by psychological distress (General Health Questionnaire-GHQ-12, score of 3 or more), [16] probable generalised anxiety (Generalised Anxiety Disorder GAD-7, score of 10 or more), [17] probable depression (Patient Health Questionnaire PHQ-9, score of 10 or more) [18] and probable post traumatic stress disorder (PTSD) (PTSD checklist-shortform, score of 14 or more) [19]. These are standard epidemiologic instruments with validated criteria indicating increased likelihood of mental health symptoms (screening tools).

### Statistical analysis

We examined the proportion of respondents who reported 'damage to property and possessions', 'disruption to services' and each of the variables that measured 'psychological exposure', according to the reported level of water in the home. The five-point Likert scale for 'psychological exposure' variables was collapsed to two categories: (i) "much better", "better" or "the same", and (ii) "worse" or "much worse", as there were very few responses in the "much better", and "better" category or in the "much worse" category.

We first examined the univariate relationship between all the exposure variables and mental health symptoms. Multivariable logistic regression was then used to describe the association between water level in the home and mental health symptoms, adjusted for the following confounding variables: age (≤ 35 yrs, 36-50 yrs, 51 + yrs), sex, presence of an existing medical condition and employment status. We included 'area' and 'method of data collection' variables in the model because of the substantial differences in the socioeconomic status between study areas and variation in data collection methods. 'Psychological exposure' variables and 'incident management' variables (evacuation and disruption to essential services) were then added as explanatory variables to this model. All data analyses were carried out using STATA version 10 [20].

## Results

### Response rates

We mailed 2,627 invitations in South Yorkshire and 8,915 invitations in Worcestershire; 2,265 people responded (38% in South Yorkshire and 14% in Worcestershire). In Worcestershire, the response rate to the postal survey was low at 12%, compared to 58% for face-to-face interviews. Among the respondents who were given the option of either a postal, telephone or web based questionnaire, 12% chose the web based questionnaire, 15% chose the telephone but the majority chose to return postal questionnaires (73%).

### Characteristics of responders

The mean age of responders was 50 yrs (s.d.17 yrs) in South Yorkshire and 57 yrs (s.d. 17 yrs) in Worcestershire, The age range for the sample was 16 yrs to 96 yrs. The age profile of responders was similar to the age profile of the sample populations based on 2001 Census data, although people aged under 36 yrs were under-represented and people over 50 yrs were over-represented. A higher proportion of females responded to the surveys (72% in South Yorkshire and 57% in Worcestershire). According to data from the Office for National Statistics (ONS) [21] 52% of the population in the sample areas were female. At least half the respondents reported that they had lived in their area for at least 15 yrs. There was also a higher proportion of responders to the survey who reported they were unemployed (28% in South Yorkshire (ONS data: 20%), 39% in Worcestershire (ONS data: 15%)) or retired (24% in South Yorkshire (ONS data: 16%), 9% in Worcestershire (ONS data: 19%).

Reported damage to property, disruption to services, health concerns relating to the flood, and perceived negative impact on finances increased in proportion to the level of flood water affecting the home (Table 1).

**Table 1:** Numbers of respondents (%) that reported damage to property, disruption to services, health concerns and perceived risk according to level of water in the home

					Perceived risk			
					
	Damage to property (%)	Disruption to essential services (%)	Asked to evacuate but refused (%)	Asked to evacuate and evacuated (%)	Reported health concerns at the time of the survey (%)	Perceived house values will be worse (%)	Perceived their finances will be worse (%)	Perceived the local environment will be worse (%)
**South Yorkshire**								

All respondents **(n = 999)**	347 (34.7)	320 (32.0)	116 (11.6)	203 (20.3)	236 (23.6)	690 (69.1)	223 (22.3)	291 (29.1)

**Water level**								

Not affected (n = 304)	3 (1.0)	47 (15.5)	12 (3.9)	12 (3.9)	31 (10.2)	198 (65.1)	29 (9.5)	77 (25.3)

Outside the house (n = 286)	30 (10.5)	85 (29.7)	39 (13.6)	44 (15.4)	45 (15.7)	194 (67.8)	35 (12.2)	81 (28.3)

Below floor level (n = 72)	41 (56.9)	23 (31.9)	14 (19.4)	13 (18.1)	19 (26.4)	54 (75.0)	24 (33.3)	25 (34.7)

Above floor level (n = 272)	265 (97.4)	158 (58.1)	51 (18.7)	128 (47.1)	132 (48.5)	228 (83.8)	126 (46.3)	99 (36.4)

**Worcestershire**								

All respondents **(n = 1267)**	169 (13.2)	125 (9.8)	18 (1.4)	46 (3.6)	138 (10.8)	624 (49.2)	162 (12.7)	270 (21.3)

**Water level**								

Not affected (n = 573)	6 (1.0)	28 (4.9)	4 (0.7)	0	41 (7.2)	259 (45.2)	38 (6.6)	119 (20.8)

Outside the house (n = 343)	15 (4.4)	36 (10.5)	1 (0.3)	5 (1.5)	36 (10.5)	182 (53.1)	39 (11.4)	77 (22.4)

Below floor level (n = 62)	32 (51.6)	13 (21.0)	2 (3.2)	8 (12.9)	10 (16.1)	46 (74.2)	15 (24.2)	17 (27.4)

Above floor level (n = 117)	114 (97.4)	43 (36.7)	11 (9.4)	33 (28.2)	38 (32.5)	100 (85.5)	60 (51.3)	35 (29.9)

### Associations between mental health symptoms, socio-demographics and water level in the home

The prevalence of all mental health symptoms was significantly higher among individuals who reported flood water in the home (psychological distress 69%, probable anxiety 48%, probable depression 43%, probable PTSD 22%) compared to individuals who did not (psychological distress 14%, probable anxiety 5%, probable depression 7%, probable PTSD 2%) (p < 0.01 for difference in proportions for each outcome).

Psychological distress, probable anxiety, probable depression and probable PTSD were 3 - 5 times higher in South Yorkshire compared to Worcestershire in univariate analysis, and were significantly raised in women, unemployed people, and in those with prior medical conditions. Water above floor level in the home was strongly associated adversely with each mental health measure, and the strength of this association changed little after adjusting for age, sex, employment, prior health status, area and data collection method (Table 2).

**Table 2:** Association between mental health outcomes and socio-demographics, previous medical problems and water level (Odds ratios and 95%CI. Adjusted odds ratios are adjusted for all variables shown in the table)

	Psychological distress (cases = 584, total = 2,113)		Anxiety (cases = 297, total = 2,037)		Depression (cases = 280, total = 2,113)		Probable PTSD (cases = 138, total = 2,019)	
Explanatory variable	Univariate	Adjusted	Univariate	Adjusted	Univariate	Adjusted	Univariate	Adjusted

**Age (years)**								

≤ 35	1.0	1.0	1.0	1.0	1.0	1.0	1.0	1.0

36-65	0.9 (0.7, 1.1)	1.1 (0.8, 1.5)	0.95 (0.7, 1.3)	1.0 (0.7, 1.6)	0.8 (0.6, 1.1)	1.0 (0.7, 1.5)	1.2 (0.7, 1.9)	1.5 (0.9, 2.5)

> 65	0.5 (0.3, 0.6)	0.6 (0.4, 1.0)	0.6 (0.4, 0.9)	0.6 (0.3, 1.1)	0.3 (0.2, 0.5)	0.4 (0.2, 0.7)	0.5 (0.2, 0.9)	0.4 (0.2, 0.9)

**Reported existing medical problems**	2.0 (1.6, 2.6)	2.4 (1.8, 3.2)	2.5 (1.8, 3.4)	2.4 (1.7, 3.5)	2.6 (1.9, 3.5)	2.9 (2.0, 4.2)	3.9 (2.6, 6.0)	4.1 (2.6, 6.7)

**Employment**								

Employed	1.0	1.0	1.0	1.0	1.0	1.0	1.0	1.0

Retired	0.75 (0.6, 0.9)	0.8 (0.5, 1.2)	1.2 (0.9, 1.6)	1.5 (0.9, 2.4)	0.9 (0.6, 1.2)	1.2 (0.8, 2.0)	1.1 (0.7, 1.7)	1.5 (0.8, 2.7)

Unemployed	2.1 (1.6, 2.7)	1.4 (1.0, 1.9)	2.2 (1.6, 3.0)	1.2 (0.8, 1.8)	2.9 (2.1, 4.0)	1.7 (1.2, 2.6)	2.8 (1.9, 4.3)	1.6 (0.9, 2.5)

**Female Gender**	1.9 (1.5, 2.3)	1.6 (1.2, 2.1)	1.7 (1.3, 2.2)	1.5 (1.0, 2.0)	2.0 (1.5, 2.7)	1.7 (1.2, 2.3)	1.9 (1.2, 2.8)	1.5 (1.0, 2.4)

**Water level**								

Not affected	1.0	1.0	1.0	1.0	1.0	1.0	1.0	1.0

Outside the house	1.8 (1.4, 2.3)	1.8 (1.3, 2.4)	2.0 (1.3, 3.1)	1.7 (1.1, 2.7)	1.2 (0.8, 1.8)	1.1 (0.7, 1.7)	2.6 (1.4, 4.8)	2.6 (1.4, 4.9)

Below floor level	3.0 (2.0, 4.6)	3.0 (1.9, 4.6)	2.4 (1.3, 4.5)	2.1 (1.1, 4.0)	3.1 (1.8, 5.3)	2.9 (1.6, 5.0)	2.8 (1.1, 7.0)	2.6 (1.0, 6.7)

Above floor level	13.4 (10.0, 17.9)	12.8 (9.3, 17.6)	17.8 (12.2, 26.0)	13.9 (9.3, 20.8)	9.5 (6.7, 13.5)	7.7 (5.2, 11.4)	14.3 (8.2, 24.9)	11.9 (6.6, 21.5)

**Area**								

Worcestershire	1.0	1.0	1.0	1.0	1.0	1.0	1.0	1.0

South Yorkshire	3.5 (2.9, 4.3)	1.5 (1.1, 1.9)	4.7 (3.5, 6.3)	1.9 (1.3, 2.8)	5.0 (3.7, 6.7)	1.9 (1.3, 2.8)	3.9 (2.7, 5.8)	1.7 (1.0, 2.8)

**Method of data collection**								

Web-based and telephone	1.0	1.0	1.0	1.0	1.0	1.0	1.0	1.0

Postal	0.6 (0.5, 0.7)	1.0 (0.7, 1.2)	0.4 (0.3, 0.6)	0.8 (0.6, 1.1)	0.5 (0.4, 0.7)	1.0 (0.7, 1.4)	0.7 (0.5, 1.0)	1.5 (1.0, 2.3)

Face to face interviews	0.2 (0.1, 0.4)	0.4 (0.2, 0.7)	0.3 (0.2, 0.5)	0.9 (0.4, 1.8)	0.1 (0.0, 0.2)	0.2 (0.1, 0.6)	0.3 (0.1, 0.7)	0.9 (0.3, 2.4)

### Associations between mental health symptoms, 'psychological exposure' and 'incident management' variables

Following adjustment for the perceived impact of the floods and evacuation variables, the odds ratios for 'water above floor level in the home' were reduced to 5.0 (95% CI 3.4, 7.3) for psychological distress, 4.8 (95% CI 3.0, 7.8) for probable anxiety, 2.6 (95% CI 1.6, 4.3) for probable depression, and 3.9 (95% CI 1.9, 7.8) for probable PTSD.

Concern that the floods would affect people's health was associated with odds ratios of 8.6 - 10.0 for the mental health symptoms in univariate analysis, and 3.0 - 4.7 following adjustment for other explanatory variables in the model. After adjustment, perception of an adverse impact on finances was associated with odds ratios of 1.8 - 3.2. (Table 3).

**Table 3:** Association between mental health outcomes and (i) psychological exposure variables and (ii) incident management activities.

	Psychological distress (cases = 584, total = 2,113)		Anxiety (cases = 297, total = 2,037)		Depression (cases = 280, total = 2,113)		Probable PTSD (cases = 138, total = 2,019)	
**Psychological exposure variables**	Univariate	Adjusted	Univariate	Adjusted	Univariate	Adjusted	Univariate	Adjusted

Reported health concerns	9.9 (7.7, 12.8)	4.7 (3.5, 6.4)	10.0 (7.5, 13.1)	3.7 (2.6, 5.1)	9.6 (7.2, 12.8)	4.3 (3.0, 6.1)	8.6 (6.0, 12.4)	3.0 (1.9, 4.7)

Perceived house values will be worse	2.3 (1.8, 2.8)	1.0 (0.7, 1.3)	3.2 (2.3, 4.4)	1.3 (0.8, 1.9)	2.5 (1.8, 3.4)	1.1 (0.7, 1.6)	2.7 (1.7, 4.4)	1.1 (0.6, 1.9)

Perceived their finances will be worse	5.3 (4.2, 6.7)	2.5 (1.8, 3.4)	4.5 (3.4, 5.9)	1.8 (1.3, 2.7)	4.4 (3.3, 5.8)	2.0 (1.3, 2.9)	6.4 (4.4, 9.3)	3.2 (2.0, 5.2)

**Incident management variables**								

Disruption to essential services	4.8 (3.8, 6.0)	1.8 (1.3, 2.4)	6.3 (4.8, 8.2)	2.1 (1.5, 3.0)	5.1 (3.9, 6.7)	2.1 (1.5, 3.0)	7.0 (4.8, 10.1)	3.1 (2.0, 4.9)

Asked to evacuate but refused	4.2 (3.0, 6.1)	1.1 (0.7, 1.8)	4.5 (3.0, 6.7)	1.0 (0.6, 1.8)	5.1 (3.3, 7.6)	1.2 (0.7, 2.1)	5.0 (3.0, 8.3)	1.0 (0.5, 1.9)

Asked to evacuate and evacuated	7.3 (5.4, 9.7)	1.7 (1.2, 2.5)	7.7 (5.7, 10.5)	1.5 (1.0, 2.3)	5.6 (4.0, 7.7)	1.2 (0.8, 1.9)	4.4 (2.9, 6.7)	0.7 (0.4, 1.2)

**Water level**								

Not affected	1.0	1.0	1.0	1.0	1.0	1.0	1.0	1.0

Outside the house	1.8 (1.4, 2.3)	1.5 (1.1, 2.0)	2.0 (1.3, 3.1)	1.3 (0.8, 2.1)	1.2 (0.8, 1.8)	0.8 (0.5, 1.3)	2.6 (1.4, 4.8)	1.8 (0.9, 3.6)

Below floor level in the lounge or kitchen or other ground floor rooms	3.0 (2.0, 4.6)	1.7 (1.0, 2.8)	2.4 (1.3, 4.5)	1.1 (0.5, 2.2)	3.1 (1.8, 5.3)	1.5 (0.8, 2.8)	2.8 (1.1, 7.0)	1.1 (0.4, 3.1)

Above floor level	13.4 (10.0, 17.9)	5.0 (3.4, 7.3)	17.8 (12.2, 26.0)	4.8 (3.0, 7.8)	9.5 (6.7, 13.5)	2.6 (1.6, 4.3)	14.3 (8.2, 24.9)	3.9 (1.9, 7.8)

(Odds ratios and 95%CI. Adjusted odds ratios are adjusted for all variables shown in the table, as well as age, previous medical problems, gender, employment, area and method of data collection)

Disruption to essential services was associated with odds ratios of 1.8 - 3.1 after adjustment. Evacuation was associated with psychological distress (OR 1.7 95% CI 1.2, 2.5), but not significantly with the other three mental health symptoms in the adjusted analysis.

## Discussion

Extreme weather with risk of flooding is predicted to increase markedly in coming years [13].

Following the severe floods in England, this public health investigation was undertaken as part of an emerging programme within the Health Protection Agency to assess the health impact of emergencies [22,23] so that in the future contributory factors can be addressed, and the impact minimised. The essential need for this type of work has since been echoed in the Government review that was commissioned to examine the lessons learnt from this UK experience [13]. As with investigations following other emergencies in England such as the Buncefield fire [24] and the London bombings [22], we used survey methods and standardised screening tools for measuring mental health symptoms. We took into account the fact that beliefs about exposure and health effects are directly linked to mental health. In the case of chemical emergencies the perception of exposure has been shown to have similar effects on distress and general symptomatology as actual exposure [25].

Our results are consistent with previous studies that reported greater mental health impact for women, and for those with prior health problems [7,10,11]. Our data suggests that the prevalence of mental health symptoms rose with the level of flood water in the home. These results are consistent with a previous smaller study in the UK, which reported a four-fold increase in psychological distress one year after the floods, comparing flooded with non-flooded households [26]. Little is known about the precipitation of psychological distress by critical incidents. We found that evacuation was associated with a modest rise in psychological distress but non-significant increases for the other three measures. In relation to chemical exposure, evacuation has previously been shown to be associated with higher morbidity [27]. Our results suggest that perceptions that the floods would have effects on health and personal finances increases mental health symptoms by two to four-fold, even when other associations are taken into account. These results are consistent with a previous UK study conducted in 2001/2 surveying householders in 30 areas flooded over the period 1998-2000, that reported "problems with insurers" was a major predictor of psychological distress and PTSD at two to four years after flooding [28]. We found that loss of essential services was a major additional risk factor, itself worsening mental health two to three fold, which emphasises the need for planners to build greater resilience into essential services. Measures to reduce ingress of water into houses is also likely to significantly reduce the psychosocial burden of flooding, since water rising above ground floor level raised adverse outcomes two to five fold. Our findings give weight to the Pitt review's recommendations to the UK Government especially in regard to household insurance (i.e. public education programmes setting out the benefits of insurance in the context of flooding), disruption of services (i.e. the need to avoid loss of essential services such as water and power) and prevention of water ingress into homes [13].

### Limitations of this study

We found that the risk of mental health symptoms was greater in South Yorkshire compared to Worcestershire. The nature of the floods was similar in these two areas but the population profiles were heterogeneous in terms of area level deprivation. South Yorkshire has a more deprived population compared to the areas surveyed in Worcestershire. Also the survey in South Yorkshire was carried out at three months after the flood compared to 6 months in Worcestershire, for logistical reasons. Further, we used different methods of data collection in conducting these surveys, which may have had different ascertainment biases. For this reason, we initially analysed the data separately for (i) South Yorkshire and Worcestershire, and (ii) data collected using face-to-face interviews and self-completed questionnaires in Worcestershire but found no differences in the associations detected and therefore we presented pooled analyses of these data in this paper, with adjustment for area and method of data collection.

Our study sample had a higher proportion of women, older people and unemployed people compared to the Office for National Statistics demographic profiles for these areas. Whilst these sample characteristics could lead to an overestimation of the prevalence of mental health symptoms in the survey areas, our main interest was in the relative rates of mental health symptoms between flooded and non-flooded groups, and we were able to adjust for these characteristics in multivariable analysis.

The response rates, although low, are typical of studies of this nature [22,24,28]. Though people with poorer mental health may have been more likely to respond than those who did not, it should be noted that the prevalence of psychological distress in individuals who were not affected by flooding was comparable to general population rates in the UK (10%-22%) [29]. In such cross-sectional studies we could not ascertain the extent to which pre-flood mental health affected post-flood mental health and individuals with pre-flood depressive symptoms may report greater increases of these symptoms post flood [30-32]. However we were able to adjust for self-reported history of health problems (respiratory, cardiac and mental health problems) prior to the floods although we were unable to validate these data against prescribing data or General Practitioner records so our results may be affected by under-reporting of mental health problems. Although we did not have data on individual measures of social class, which is a known risk factor for psychological distress, we were able to adjust for employment status (employed, unemployed, retired) in our analysis.

## Conclusion

Despite the inherent methodological difficulties in epidemiological assessment of acute incidents we have quantified the psychosocial impacts. Strategies for minimising this impact need to be developed and built in to emergency preparedness and response systems [13,33]. Reporting health concerns and perceived negative impact on finances were independent predictors of psychological distress highlighting the need to respond to the public's concerns following such incidents, addressing perceived difficulties and hazards as well as objectively verifiable exposures. Individuals who were evacuated from their homes were also at higher risk of psychological distress. Public Health Agencies should consider these risk factors in planning emergency preparedness, ensuring that evacuation requests are only made when essential and non-evacuation strategies explored, that financial issues relating to the flood are addressed, in addition to addressing health concerns when providing information and advice to people affected by flooding, and that disruptions to essential services are avoided or minimised as far as possible. Further work is required to investigate risk factors associated with psychological distress in individuals who are evacuated from their homes and effective interventions to minimise the impact of emergencies on communities. Public health agencies need to develop and evaluate strategies for improved risk communication and psychological support for flooded families.

## Competing interests

The authors declare that they have no competing interests.

## Authors' contributions

SRP initiated and led the investigation. All authors contributed to the conception and design of the study. SP and JG analysed the data and SP wrote the first draft of the manuscript. All authors contributed to interpretation of the data and critical revision of the manuscript. SRP is the guarantor for this study.

## Pre-publication history

The pre-publication history for this paper can be accessed here:

http://www.biomedcentral.com/1471-2458/11/145/prepub
